# Enterovirus 71 infection of human airway organoids reveals VP1-145 as a viral infectivity determinant

**DOI:** 10.1038/s41426-018-0077-2

**Published:** 2018-05-09

**Authors:** Sabine M. G. van der Sanden, Norman Sachs, Sylvie M. Koekkoek, Gerrit Koen, Dasja Pajkrt, Hans Clevers, Katja C. Wolthers

**Affiliations:** 1 Department of Medical Microbiology, Academic Medical Center, 1105 AZ Amsterdam, The Netherlands; 2 Hubrecht Institute, Royal Netherlands Academy of Arts and Sciences (KNAW), University Medical Center Utrecht and Cancer Genomics Netherlands, 3584 CT Utrecht, The Netherlands; 3 Department of Pediatric Infectious Diseases, Emma Children’s Hospital, Academic Medical Center, 1105 AZ Amsterdam, The Netherlands

## Abstract

Human enteroviruses frequently cause severe diseases in children. Human enteroviruses are transmitted via the fecal–oral route and respiratory droplets, and primary replication occurs in the gastro-intestinal and respiratory tracts; however, how enteroviruses infect these sites is largely unknown. Human intestinal organoids have recently proven to be valuable tools for studying enterovirus–host interactions in the intestinal tract. In this study, we demonstrated the susceptibility of a newly developed human airway organoid model for enterovirus 71 (EV71) infection. We showed for the first time in a human physiological model that EV71 replication kinetics are strain-dependent. A glutamine at position 145 of the VP1 capsid protein was identified as a key determinant of infectivity, and residues VP1-98K and VP1-104D were identified as potential infectivity markers. The results from this study provide new insights into EV71 infectivity in the human airway epithelia and demonstrate the value of organoid technology for virus research.

## Introduction

*Picornaviridae* family enteroviruses are important human pathogens causing a broad spectrum of disease symptoms, ranging from diarrhea and skin rashes to more severe diseases, such as meningitis and paralysis^1,2^. Enteroviruses are mainly transmitted via the fecal–oral route and replicate in the gastro-intestinal tract. Alternatively, enteroviruses may spread via respiratory droplets and replicate in the respiratory tract. From both sites, enteroviruses can spread to the bloodstream and infect secondary target tissues, such as the central nervous system (CNS), often leading to increased disease severity. Understanding enterovirus–host interactions at their primary replication sites is therefore essential for developing preventive and therapeutic strategies; however, it is largely unknown how enteroviruses infect these sites.

Along with the polioviruses, enterovirus 71 (EV71) is among the enteroviruses whose pathogenesis has been studied most extensively. While the polioviruses are nearing extinction, EV71 has caused large outbreaks of hand, foot, and mouth disease and more severe neurologic diseases, including brainstem encephalitis^3,4^. The increased incidence of EV71 infections is associated with the rapid emergence of new subgenotypes, defined by their nucleotide diversity in the VP1 capsid protein. To date, seven genogroups (A–G), with multiple subgenotypes in genogroup B (numbered B0–B5) and C (C1–C5) have been identified^5,6^. Only strain- but no genotype-dependent differences in the disease outcome severity have been described^7–9^.

Although the gastro-intestinal tract is generally accepted as the main site of primary EV71 replication, detecting viral RNA and infectious viruses in respiratory samples of EV71-infected patients indicates that the virus also replicates in the respiratory tract^3,10–12^. Furthermore, pulmonary edema with pathogenic lesions in the lungs is commonly observed among fatal cases of EV71 infection^3^. It is unclear whether this is a consequence of brainstem encephalitis or viral replication in lung tissue. In rhesus monkeys, EV71 replicated more efficiently in the airways, particularly in the lungs and bronchial tubes, than in the digestive tract, suggesting that the virus is primarily respiratory tract tropic^13^. In addition, infection via the respiratory tract resulted in more severe neurologic diseases, suggesting a relationship between the primary EV71 replication site and the disease outcome severity. The main EV71 receptor, the lysosomal membrane protein, Scavenger Receptor Class B Member 2 (SCARB2), is abundantly expressed in both the human intestinal and respiratory tracts^14,15^.

EV71–host interaction studies on the entry sites have been hampered by the lack of suitable models mimicking human disease development. For example, a commonly used hSCARB2 transgenic mouse model shows EV71 replication in CNS tissue but not in the gastro- intestinal or respiratory tracts^9^. Furthermore, virulence markers identified in in vitro and in vivo experiments often contradict those identified from molecular epidemiological data. For example, in humans, a glutamine (Q) at VP1-145 has been associated with increased disease severity based on complete genome analyses of clinical isolates, whereas studies in cynomolgus monkeys and mice identified a glutamic acid (E) as a marker of increased (neuro)virulence^16–19^. This same residue plays a role in determining binding to the second identified receptor P-selectin glycoprotein ligand 1 (PSGL1), which is expressed on white blood cells^20^. However, both PSGL1-binding strains (VP1-145G/Q) and non-binding strains (VP1-145E) have been isolated from mild and severe cases, suggesting that VP1-145 plays another, yet unknown, role in determining disease outcome severity.

The development of human three-dimensional (3D) culture models (organoids) that closely resemble the complex multicellular composition and physiology of human tissues in vivo has created the opportunity to study virus–host interactions in a human setting^21^. These models have successfully been used to amplify previously unculturable viruses from clinical specimens (e.g., norovirus in human intestinal organoids) and to study both Zika virus (using human forebrain organoids) and rotavirus (using human intestinal organoids) pathogenesis^22–24^. Enterovirus infection studies in human intestinal organoids have recently revealed novel insights on enterovirus–host interactions in the human intestinal tract^25^. Enterovirus infections induced virus-type-dependent antiviral and inflammatory responses in the intestinal organoids. These responses remained absent in an immortalized intestinal cell line, illustrating that human organoid models resemble in vivo processes more closely than immortalized/cancer cell lines. A 3D organoid culture model of the human airway epithelium was recently developed. Airway basal stem cells derived from digested lung tissue can be cultured into epithelial structures surrounding a luminal cavity in a laminin and collagen-rich gel. The stem cell-derived organoids recapitulate hallmarks of the human differentiated airway epithelium, including cell-type composition (basal, club, goblet, and ciliated cells), ciliary movement, and mucus production (Sachs et al. manuscript submitted). Transcriptome analyses of airway organoids derived from several donors have confirmed abundant EV71 receptor SCARB2 expression, while PSGL1 expression was absent (Sachs et al. unpublished data). As the respiratory tract represents a primary EV71 replication site, we assessed whether these organoids are susceptible to infection with EV71 clinical isolates and form a relevant model for EV71–host interaction studies in the human airway.

## Results

### EV71 strain-dependent replication kinetics in human airway organoids

**Fig. 1:**
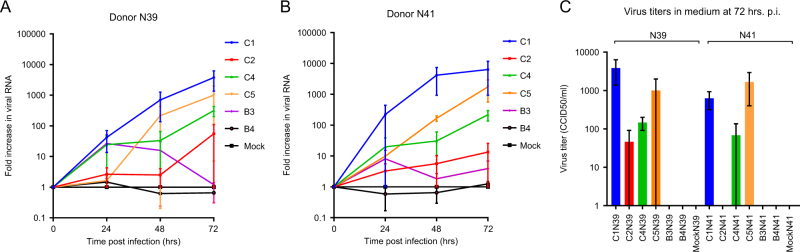
Replication kinetics of enterovirus 71 (EV71) in human airway organoids. **a**, **b** Fold increases in viral RNA of EV71 subgenotype strains C1 (C1 91-480), C2 (2485), C4 (75-Yamagata), C5 (209-VN), B3 (SK-EV006), and B4 (C7-Osaka) in donors N39 and N41, determined by RT-PCR on lysed organoids. Data present the mean fold increase in two infection experiments + standard error of the mean (SEM). **c** Viral titers detected in the medium covering the gel-embedded organoids at 72 h postinfection (p.i.). Titers are expressed as the mean 50% cell culture infective dose (CCID_50_)/ml in two infection experiments + SEM

To assess the susceptibility of human airway organoids to EV71 infection, normal organoids from two unrelated adult donors (N39 and N41) were infected with clinical isolates representing six EV71 subgenotypes (B3, B4, C1, C2, C4, and C5). Real-time PCR assays on lysed organoids from both donors consistently showed the highest fold increases in viral RNA for the C1 strain, indicating that this strain replicated the most efficiently (Fig. 1a, b). The detected 50% cell culture infectious dose (CCID_50_) in the medium covering the embedded organoids at 72 h postinfection (p.i.) confirmed that infectious C1 virus particles were produced and shed (mean titers of 3843 and 624 CCID_50_/ml in donors N39 and N41, respectively) (Fig. 1c). The C4 strain consistently replicated at intermediate levels, which was consistent with viral titers detected in the medium (mean titers of 146 and 68 CCID_50_/ml at 72 h p.i., respectively). The C5 strain efficiently replicated in donor N41, but it replicated in only one of the two infection experiments in donor N39 (7- vs 1000-fold increase in N39 at 72 h p.i.). The C2, B3, and B4 strains generally replicated poorly (averages of <2.5× increase in RNA level and no detectable titers in the medium), except one C2 infection experiment in donor N39, in which a 110-fold increase in viral RNA and a titer of 91.2 CCID_50_ were detected (Fig. 1c). Overall, the results indicated an EV71 strain-dependent level of infectivity of the airway organoids, which was consistent in two donors.

### Capsid amino acid sequence analysis revealed VP1-145 as a potential infectivity determinant

**Fig. 2:**
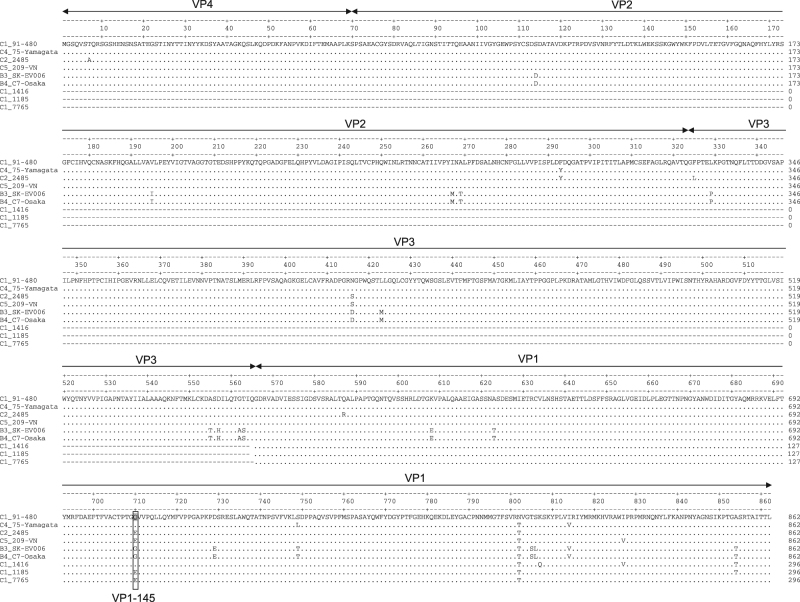
Amino acid sequence comparison of the EV71 strain capsid regions included in the current study. Residue 145 of the VP1 capsid region is marked by a box

To identify genetic infectivity determinants in the EV71 capsid proteins, complete capsid amino acid sequences of the strains included in this study were analyzed. Residue VP1-145, located in the surface-exposed DE loop and previously identified as a virulence marker in animal models and humans, differed among strains that consistently replicated in the organoids (VP1-145Q in the C1 and C4 strains) vs strains that replicated poorly (VP1-145G in B3 and B4) and strains that varied in infection efficiency among biological replicates (VP1-145E in C2 and C5) (Fig. 2).

**Fig. 3:**
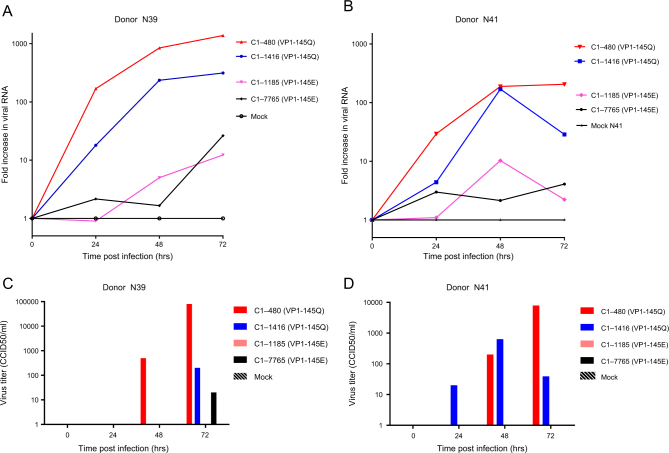
Replication kinetics of EV71 subgenotype C1 strains with a glutamine (Q) or glutamic acid (E) at VP1 residue 145 in human airway organoids. **a**, **b** Fold increases in viral RNA of EV71 strains in donors N39 and N41, respectively. Values presented were calculated from RT-PCR assays of lysed organoids. **c**, **d** Viral titers detected in the medium covering embedded organoids of donors N39 and N41, respectively, at 0 to 72 h postinfection. Titers are expressed as the 50% cell culture infective dose (CCID_50_)/ml

To verify VP1-145’s role in determining infectivity, airway organoids from the two donors were subsequently infected with EV71 subgenotype C1 VP1-145E or VP1-145Q clinical isolates (VP1 sequences are presented in Fig. 2). The VP1-145Q clinical isolates replicated more efficiently than did the VP1-145E isolates (mean fold increase of 482× vs 11× at 72 h p.i.; Fig. 3a, b). This was consistent with viral titers detected in the medium (mean titers of 21,903 vs 5 CCID_50_/ml at 72 h p.i., respectively; Fig. 3c, d).

### Confirming VP1-145 as an infectivity determinant by site-directed mutagenesis

**Fig. 4:**
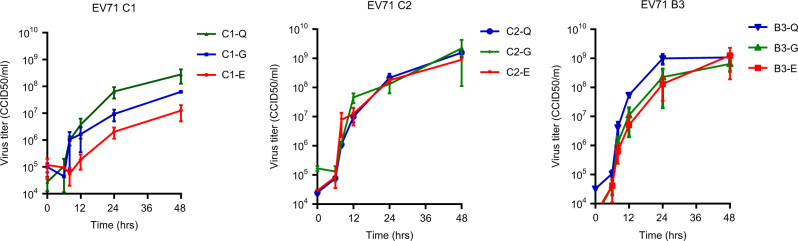
Growth characteristics of EV71 C1, C2, and B3 strains with a glutamic acid (E), glycine (G), or glutamine (Q) at VP1-145, generated by site-directed mutagenesis, in rhabdomyosarcoma (RD) cells. Data represent the mean 50% cell culture infective dose (CCID_50_)/ml in two infection experiments + SEM

**Fig. 5:**
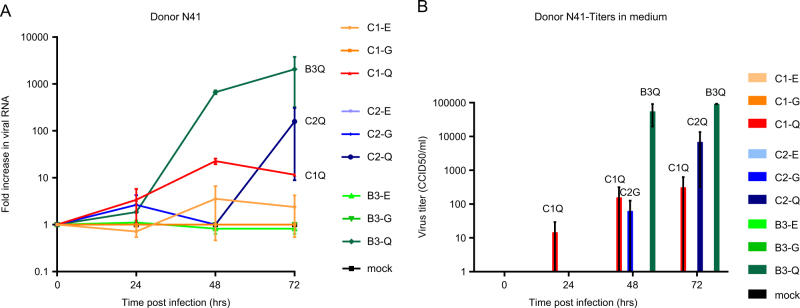
Replication kinetics of EV71 strains with a glutamic acid (E), glycine (G), or glutamine (Q) at VP1-145, generated by site-directed mutagenesis, in human airway organoids. **a** Fold increases in viral RNA of C1, C2, and B3 strains with VP1-145E, -G, or -Q in donor N41, determined by RT-PCR assay on lysed organoids. Data present the mean fold increase in two infection experiments + SEM. **b** Viral titers detected in the medium covering embedded organoids at 0–72 h postinfection. Titers are expressed as the mean 50% cell culture infective dose (CCID_50_)/ml in two infection experiments+SEM

To verify VP1-145 as an infectivity determinant, we generated viral particles (representing EV71 subgenotypes, B3, C1, and C2) with a VP1-145Q, VP1-145E, or VP1-145G by site-directed mutagenesis. The complete capsid-encoding regions were sequenced to verify the presence of the correct VP1-145 residue and to exclude residue mutations elsewhere in the capsid region. All viral mutants showed a productive infection in rhabdomyosarcoma (RD) cells (Fig. 4). C1 VP1-145E and VP1-145G mutant titers averaged 22× and 4× lower, respectively, than those of the C1 VP1-145Q mutant at 48 h p.i. (average titer of 10^8.4^ CCID_50_/ml), whereas C2 and B3 mutant titers differed by ≤2.5-fold (average titers of 10^9.2^ and 10^9.0^ CCID_50_/ml, respectively). Donor N41 airway organoid infection with the mutant viruses showed that VP1-145Q variants of the three subgenotypes consistently replicated more efficiently than the VP1-145E and VP1-145G variants (mean fold increases of 12×, 159× and 2055× for C1, C2, and B3 VP1-145Q mutants, respectively, vs 1.4× for VP1-145E and G variants) (Fig. 5a). Except for a C2 VP1-145G mutant in one of the two infections, infectious viral particle shedding in the medium was only detected for VP1-145Q variants (average titers of 10^2.5^, 10^3.8^, and 10^5.0^ CCID_50_/ml for C1, C2, and B3, respectively), confirming that VP1-145Q is a key determinant of increased infectivity in airway organoids (Fig. 5b).

### Identifying additional infectivity markers

**Fig. 6:**
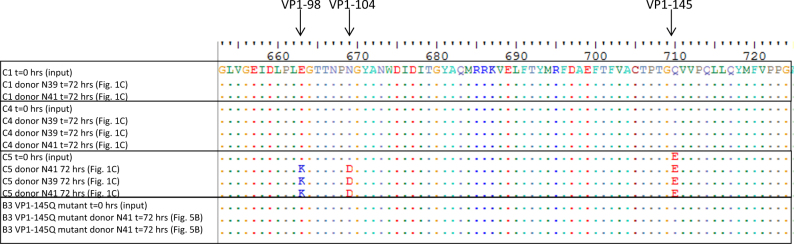
**Comparison of EV71 VP1 amino acid sequences that were retained from the organoid culture medium 72 h postinfection**

To determine whether mutating VP1-145 could explain the occasionally successful C2 and C5 strain replication, organoid medium samples that were positive in titration assays were used to retain the VP1 capsid sequences. The VP1 sequences were successfully obtained from the three C5-positive medium samples, but not from the C2-positive sample, collected from both donors at 72 h p.i. (Fig. 1c). In all three C5 strains, VP1 residues, including residue VP1-145, remained unchanged (VP1-145E), except for two in the neighboring BC-loop, E98K, and N104D (Fig. 6). These mutations did not occur in the C1 and C4 clinical isolates or the B3-145Q mutant viruses collected from both donors at 72 h p.i. The relatively high replication kinetics of C5 in the absence of VP1-145Q suggest that VP1-98K and VP1-104D are two additional infectivity determinants.

## Discussion

In this study, we demonstrated the susceptibility of a newly developed human airway organoid model for EV71 infection and showed for the first time in a human physiological model that EV71 replication kinetics are strain-dependent.

Increased replication capacities of EV71 strains have previously been associated with increased disease severity in animal models, and positive correlations have been found between respiratory virus titers in the respiratory tract and disease severity in humans^8,26,27^. Importantly, identifying VP1-145Q as a marker of increased infectivity in the organoids is consistent with previous observations that VP1-145Q variants are associated with increased disease severity in humans^16,18^. In contrast, animal model studies identified VP1-145E as a marker of increased virulence^17,19^. Since VP1-145E is critical for mouse adaptation, studying pathogenesis of naturally occurring VP1-145 variants, such as VP1-145Q (representing ~9% of circulating human strains), is complicated in non-transgenic murine models^17,20^. In cynomolgus monkeys, VP1-145’s role in EV71 pathogenesis has only been evaluated in VP1-145E and G variants^19^. The observed neurovirulent character of the VP1-145E strain in this model could potentially be explained by the presence of VP1-98K, which we identified as a potential marker of increased infectivity in the airway organoids.

Using organoids in viral research requires further standardization, as illustrated by the intra- and inter-donor variation observed among the biological infection replicates in the current study. Nevertheless, the overall observed trends in strain-dependent infectivity were similar in organoids from two unrelated donors, which underlines the validity of our results. Strain-dependent replication kinetics have previously been observed in immortalized/cancer cell lines, and several viral markers of cell culture growth characteristics have been identified^28–30^. However, these virulence determinants were mostly identified in the 5’-untranslated region (5’UTR) and the non-structural proteins, playing a role in viral replication, translation, and intracellular interactions with the host. Using RD and Jurkat cells, Nishimura et al. (2013) demonstrated that VP1-145 acts as a molecular switch to control PSGL1 receptor binding^20^. However, they also demonstrated that VP1-145E/G/Q variants all replicated well in RD cells, which is consistent with the VP1-145 mutant virus growth curves presented in Fig. 4 of the current study. VP1-145’s role in determining disease outcome has mostly been identified using animal models and epidemiologic molecular data, and therefore identifying this determinant using human organoids emphasizes the relevance of using organoid models for virus–host interaction studies.

The underlying mechanism of VP1-145, and potentially those of VP1-98 and -104, in determining airway site infectivity remains unclear. Of interest, residues VP1-145 and -98 have been reported to play critical roles in mouse adaptation by improving EV71 binding to mouse SCARB2^17,31^. This could suggest that the strain-dependent infectivity observed in airway organoids is related to human SCARB2 binding. Furthermore, positively charged residues at VP1-98 and VP1-145 have recently been reported to modulate EV71 binding to its attachment receptor, heparan sulfate^32^. VP1-98E-145Q strains were shown to have strong heparan sulfate-binding phenotypes, and mutation of VP1-98E to K could fully restore the heparan sulfate-binding properties of VP1-98E-145E variants. The higher infectivity of VP1-98E-145Q variants and the VP1-98K-145E C5 variant in the airway organoid model compared to the VP1-98E-145E/G viruses could therefore also be attributed to a heparan sulfate-binding phenotype.

A role of the second identified EV71 receptor, PSGL1, which binds EV71 strains with either a VP1-145G or Q, is unexpected, as this receptor is mainly expressed on lymphocytes^14,33^. Transcriptome analyses of airway organoids derived from several donors confirmed the absence of PSGL1, whereas SCARB2 was abundantly expressed (data not shown). Extended studies are required to verify the roles of SCARB2 and co-receptors, such as heparan sulfate, in EV71 infection of the airway organoid model and to unravel the exact functions of VP1-145 (and VP1-98 and -104) in determining infectivity.

This study focused on identifying infectivity markers in the capsid region; however, this does not exclude the possibility that additional (yet unidentified) virulence markers are located outside the capsid region. The superior replication of B3 VP1-145Q mutants compared with C1 and C2 VP1-145Q mutants points to this possibility. Virulence markers have previously been identified in the 5’UTR and non-structural encoding regions of EV71^34,35^; however, complete genome sequence analysis of the C1, C2, B3, and B4 clinical isolates and mutant viruses included in this study revealed no correlation between replication kinetic differences and polymorphism of these markers.

In addition to viral markers, genetic host factors have been reported to play roles in infectivity, potentially explaining why massive outbreaks are restricted to the Asian region^36–38^. No differences in donor-dependent susceptibility were observed in the current study; however, this might be a consequence of including only organoids from two Caucasian donors.

Using a recently developed human airway organoid model, we identified viral factors that determine EV71 infectivity in the human airway epithelium. Genetic heterogeneity of these markers and associated differences in replication capacity may explain differences in disease severity in EV71-infected children. This successful application of airway organoids in identifying these markers paves the way for extended studies on EV71 pathogenesis and that of other enteroviruses in the human airway epithelium.

## Materials and Methods

### Cells and viruses

Viral strains used in the current study were amplified in RD and human colorectal adenocarcinoma (HT-29) cells (American Type Culture Collection, ATCC). Cell lines were cultured at 37 °C and 5% CO_2_ in Eagle’s Minimum Essential medium (EMEM, Lonza) supplemented with 10% fetal bovine serum (FBS, Sigma-Aldrich), 100 IU/ml of penicillin, and 100 µg/ml of streptomycin. EV71 C1 strains 480, 7765, 1416, and 1185 and C2 07-2485 were isolated from clinical specimens as part of the national enterovirus surveillance system in the Netherlands between 1991 and 2007^39^. Strains isolated from outbreaks in Asia originated from Malaysia (B3 SK-EV006, 1997), Japan (B4 C7-Osaka, 1997; C4 75-Yamagata, 2003), and Vietnam (C5 209-VN, 2006).

### Primary human airway organoid cultures

Patient data and donor tissue for generating airway organoids were collected per the guidelines of the European Network of Research Ethics Committees (EUREC) following European, national, and local laws. The medical ethical committees of the UMC Utrecht and St. Antonius Hospital Nieuwegein approved protocols TCBio 15-159 and Z-12.55, respectively, and all patients signed informed consent approved by the responsible authority. All human organoid lines used in this study were generated from anonymized adult patient tissues from St. Antonius Hospital Nieuwegein and are part of the lung biobank of Hubrecht Organoid Technology.

Normal airway organoids were generated from normal and tumor organoids from resected normal surplus lung tissue of non-small cell lung cancer patients (lower airways and lung parenchyma). Airway organoids were cultured as previously described (Sachs et al., manuscript submitted). Briefly, organoids were established from digested lung tissue embedded in 10 mg/ml cold Cultrex growth factor-reduced Basement Membrane Extract (BME) type 2 (Trevigen-3533-010-02). Forty µl drops of BME cell suspension were solidified on prewarmed 24-well suspension culture plates (Greiner-M9312) at 37 °C for 10–20 min. Solidified BME droplets were overlaid with 400 µl of airway organoid medium (Sachs et al., manuscript submitted). Organoids were grown in humidified 37 °C/5% CO_2_ incubators at ambient O_2_. The medium was changed every 4 days, and organoids were passaged every 2 weeks by mechanical disruption through flamed glass Pasteur pipettes.

### Human airway organoid infection

Human airway organoids cultured for 14 days were removed from the gel and opened by pipetting vigorously approximately ten times to allow apical exposure to the virus. Split organoids were equally distributed into 24-well plate wells (approximately 5000 organoids/well) and incubated with 4000 CCID_50_ in EMEM supplemented with 2% FBS, 100 IU/ml of penicillin, 100 µg/ml of streptomycin, and 10 μM Rock inhibitor at 37 °C and 5% CO_2_ for 4 h. Organoids were resuspended hourly. After 4 h, the organoids were washed twice in phosphate-buffered saline (PBS) and once in Advanced Dulbecco’s modified Eagle’s medium/F-12 (Life Technologies) and resuspended in 160 µl of gel. The gel was equally distributed into four wells (for four sampling time points) of a 24-well plate and covered with 500 μl of airway organoid culture medium. All medium was collected from one well directly after embedding (*t* = 0 h) and at *t* = 24, 48, and 72 h p.i. At these same time points, organoids were harvested in 350 µl of lysis buffer and stored with the medium samples at −80 °C.

### Viral RNA detection by real-time PCR

Total RNA was extracted from the lysed organoids using the GenElute™ Mammalian Total RNA Miniprep Kit (Sigma-Aldrich) per the manufacturer’s instructions. Viral RNA was eluted in 50 µl of elution buffer. cDNA and EV-specific real-time PCR reactions were prepared as described previously^40^. In the lysed organoid samples, EV Ct values were normalized against those of the β-actin gene and used to calculate the fold increase in viral RNA relative to *t* = 0 h using the ΔΔCt method^41^.

### Quantification of infectious viruses

Infectious viral particle production was analyzed by determining the CCID_50_ in the culture medium by end-point dilution. Briefly, in a 96-well format, six replicates of a tenfold serial dilution of the medium samples were incubated with RD cells in EMEM supplemented with 10% FBS at 37 °C and 5% CO_2_ for 5 days. The CCID_50_ was calculated by scoring the cytopathic effect (CPE) in all wells using the Spearman–Kärber formula^42^.

### Capsid sequence analysis

Viral RNA was extracted from 50 µl of EV71 viral stocks and 100 µl of organoid medium samples using the GenElute™ Mammalian Total RNA Miniprep Kit (Sigma-Aldrich) per the manufacturer’s instructions. Viral RNA was eluted in 50 µl of elution buffer. The capsid-encoding regions were PCR amplified in four overlapping regions using specific primers for genogroups B and C and a PCR amplification protocol described previously^43^. PCR products were sequenced using the ABI Prism BigDye Terminator Cycle Sequencing Ready Reaction Kit, version 3.2 (Applied Biosystems, Foster City, CA, USA) on an automated sequencer (Applied Biosystems model 3700). Sequence data were edited and capsid region consensus sequences were generated by assembling overlapping DNA sequences determined from both strands using the Clustal W method implemented in BioEdit (version 7.2.5)^44^.

### Mutant virus generation

Previously constructed full-length genomic cDNA clones of three clinical isolates (C1 02363, C2 1095, and B3 SK-EV006) were used to introduce amino acid mutations at VP1-145 by site-directed mutagenesis using PCR^20^. The primers used for the mutagenesis are listed in Table S1. Linearized clones were transcribed with T7 RNA polymerase (MEGAscript T7 Kit, Ambion) per the manufacturer’s protocol. The RNA was transfected into RD cells using a Lipofectamine 2000 reagent (Life Technologies) per the manufacturer’s instructions. Cells were incubated at 37 °C and 5% CO_2_ until a clear CPE was visible. Cells were freeze–thawed three times to release viral particles, and viruses were amplified once more in fresh RD cells. The infectious viral particle amounts in the supernatants were determined as described above, and mutation introduction was verified by sequencing complete capsid-encoding regions. To characterize mutant viral growth, RD cells were grown to 90% confluency in 96-well plates and infected with the mutant viruses at a multiplicity of infection of 0.3 for 30 min at 37 °C and 5% CO_2_. Infected cells were subsequently washed twice with PBS and incubated at 37 °C and 5% CO_2_ in 100 µl of EMEM supplemented with 10% fetal calf serum. Cells were frozen at 0, 6, 8, 12, 24, and 48 h p.i., and viral titers at each time point were determined by virus titration assays as described above.

## Electronic supplementary material

Supplemental Table S1
