# Supplementary material for: Enterovirus 71 infection of human airway organoids reveals VP1-145 as a viral infectivity determinant
Source: Emerg Microbes Infect. 2018 May 9;7:84. doi: 10.1038/s41426-018-0077-2 (PMC5943241; doi:10.1038/s41426-018-0077-2)
Supplement: Supplementary file 1 — Supplemental Table S1 [file 41426_2018_77_MOESM1_ESM.doc]

**Supplemental information**

**Table S1:**

| Variant* | Sequence of forward (FW) and reverse (Rev) primer** (5’-3’) |
| --- | --- |
| C1 VP1-145G | FW: GCACACCCACCGGGGGGGTTGTCCCACAAT REV: ATTGTGGGACAACCCCCCCGGTGGGTGTGC |
| C1 VP1-145Q | FW: TGCACACCCACCGGGCAGGTTGTCCCACAA REV: TTGTGGGACAACCTGCCCGGTGGGTGTGCA |
| C2 VP1-145E | FW: GCACGCCTACCGGGGAGGTTGTTCCGCAAT REV: ATTGCGGAACAACCTCCCCGGTAGGCGTGC |
| C2 VP1-145Q | FW: GCACGCCTACCGGGCAGGTTGTTCCGCAAT REV: ATTGCGGAACAACCTGCCCGGTAGGCGTGC |
| B3 VP1-145E | FW: GCACTCCCACCGGCGAGGTTGTTCCACAAT REV: ATTGTGGAACAACCTCGCCGGTGGGAGTGC |
| B3 VP1-145Q | FW: GCACTCCCACCGGCCAGGTTGTTCCACAAT REV: ATTGTGGAACAACCTGGCCGGTGGGAGTGC |

* The original infectious clones of C1, C2 and B3 contained VP1-145E, -G and -G, resp.
**Targeting nucleotides 419-448 of the VP1 capsid protein (reference strain: C1 91-480).
